# Novel Insights into Mitochondrial DNA: Mitochondrial Microproteins and mtDNA Variants Modulate Athletic Performance and Age-Related Diseases

**DOI:** 10.3390/genes14020286

**Published:** 2023-01-21

**Authors:** Hiroshi Kumagai, Brendan Miller, Su-Jeong Kim, Naphada Leelaprachakul, Naoki Kikuchi, Kelvin Yen, Pinchas Cohen

**Affiliations:** 1 The Leonard Davis School of Gerontology, University of Southern California, Los Angeles, CA 90089, USA; 2 Graduate School of Health and Sport Science, Nippon Sport Science University, Setagaya-ku, Tokyo 158-8508, Japan

**Keywords:** small open reading frame, microprotein, mitochondrial-derived peptide, MOTS-c, genetic polymorphism, athletic performance, injury, age-related diseases

## Abstract

Sports genetics research began in the late 1990s and over 200 variants have been reported as athletic performance- and sports injuries-related genetic polymorphisms. Genetic polymorphisms in the α-actinin-3 (ACTN3) and angiotensin-converting enzyme (ACE) genes are well-established for athletic performance, while collagen-, inflammation-, and estrogen-related genetic polymorphisms are reported as genetic markers for sports injuries. Although the Human Genome Project was completed in the early 2000s, recent studies have discovered previously unannotated microproteins encoded in small open reading frames. Mitochondrial microproteins (also called mitochondrial-derived peptides) are encoded in the mtDNA, and ten mitochondrial microproteins, such as humanin, MOTS-c (mitochondrial ORF of the 12S rRNA type-c), SHLPs 1–6 (small humanin-like peptides 1 to 6), SHMOOSE (Small Human Mitochondrial ORF Over SErine tRNA), and Gau (gene antisense ubiquitous in mtDNAs) have been identified to date. Some of those microproteins have crucial roles in human biology by regulating mitochondrial function, and those, including those to be discovered in the future, could contribute to a better understanding of human biology. This review describes a basic concept of mitochondrial microproteins and discusses recent findings about the potential roles of mitochondrial microproteins in athletic performance as well as age-related diseases.

## 1. Introduction

The human genome is composed of nuclear and mitochondrial genomes. The human nuclear genome consists of twenty-two autosomes and two sex chromosomes, which contain approximately 3.3 billion DNA base pairs, while the mitochondrial genome (mtDNA) is a circular DNA molecule containing 16,569 DNA base pairs. Differences in the DNA sequence are called genetic polymorphism and there are several types of polymorphism: single-nucleotide polymorphism (SNP), insertion/deletion, and copy number variation. These polymorphisms partially or largely influence human phenotypes, such as height, weight, intelligence, personality, susceptibilities to diseases, and other traits. Human traits are influenced by both environmental and genetic factors, and heritability represents a contribution of the genetic factors to the particular trait.

The heritability estimate of athlete status is calculated at around 66%, which means 66% of the variance in athletic performance is explained by genetic factors while the remaining 34% is explained by environmental factors [1]. Furthermore, the heritabilities of athletic performance-related phenotypes are relatively high; 45–99.5% for skeletal muscle fiber composition [2,3], 49–56% for muscular strength [4], and 44–68% for cardiorespiratory fitness [5]. In addition to these physiological phenotypes, an estimated heritability of injury is 60–80% [6]. These data suggest that unrevealed genetic factors play crucial roles in determining athletic performance, and research about sports genetics to identify the detailed genetic factors began in the late 1990s [7,8,9]. To date, over two hundred genetic polymorphisms encoded in both the nuclear DNA and mtDNA were identified to be associated with athlete status [10].

Although the Human Genome Project was launched in 1990 and completed in 2003, recent studies are unraveling novel concepts hidden in the human genome: microRNA, long non-coding RNA, and small open reading frames (smORFs). Open reading frames (ORFs) are defined as DNA sequences between the start and stop codons and the term smORF is used for the ORFs with less than 100 codons that are actually translated [11,12]. Indeed, recent studies have demonstrated the existence of previously unannotated microproteins translated from smORFs on both nuclear and mitochondrial genomes. Among them, ten mitochondrial microproteins (also called mitochondrial-derived peptides: MDPs), such as humanin [13,14,15], MOTS-c (mitochondrial ORF of the 12S rRNA type-c) [16], SHLPs 1–6 (small humanin-like peptides 1 to 6) [17], SHMOOSE (Small Human Mitochondrial ORF Over SErine tRNA) [18], and Gau (gene antisense ubiquitous in mtDNAs) [19] have been identified.

Those microproteins have the potential to give us a better understanding of human biology. For example, genetic variants in the mitochondrial microproteins humanin and SHMOOSE coding region are associated with Alzheimer’s disease and cognitive decline [18,20], while that of the MOTS-c coding region is associated with type 2 diabetes risk, visceral fat area, and skeletal muscle property [21,22]. Since mitochondrial microproteins are derived from mitochondria, those are expected to have crucial roles in organs with high mitochondrial content, such as in skeletal muscle. However, little is known about the roles of mitochondrial microproteins in skeletal muscle.

In this review, we will summarize the findings about athletic performance-related genetic polymorphisms in both nuclear DNA and mtDNA, describe a basic concept of mitochondrial microproteins, and discuss the potential roles of mitochondrial microproteins in athletic performance and age-related diseases.

## 2. Nuclear Genome Encoded Genetic Polymorphism in Athletes

### 2.1. Athletic Performance-Related SNPs

A large part of the reported genetic polymorphisms is encoded in the nuclear DNA, and the R577X polymorphism (rs1815739) in the α-actinin-3 gene (ACTN3) is one of the most studied genetic markers for athletic performance beyond ethnicity. The α-actinin-3 protein, a major component of the Z-line in the skeletal muscle, is expressed in fast-twitch fibers and the *ACTN3* R577X polymorphism causes α-actinin-3 deficiency [23]. The X allele carriers of the *ACTN3* polymorphism show reduced fast-twitch muscle fibers by regulating calcineurin signaling and exhibit lowered muscle mass and strength [24,25,26]. Thus, the connection between the *ACTN3* polymorphism and athletic performance was observed in several sports events, such as track and field [9,27,28,29,30], ball games (i.e., soccer, basketball, volleyball, and handball) [31,32], martial arts [32], and gymnastics [33,34].

Another well-studied genetic polymorphism is an angiotensin-converting enzyme (*ACE*) I/D (rs4341). This *ACE* I/D polymorphism was the first genetic marker associated with physical performance [7,8]: Caucasians with the I and D alleles are associated with high endurance and sprint/power performance, respectively [35,36,37]. On the other hand, the association between the *ACE* I/D polymorphism and athletic performance may be the opposite in the Asian population. Several studies in sprint/power athletes, marathon runners, and swimmers demonstrated that the I and D alleles were associated with sprint/power and endurance performance, respectively [38,39,40]. Furthermore, it has been reported that Japanese males with the D allele exhibited higher slow-twitch fibers than those with the II genotype [25]. These results suggest that the influence of the *ACE* I/D polymorphism on athletic performance is different among ethnicities.

### 2.2. Genome-Wide Association Study (GWAS) for Athletic Performance

GWAS is an unbiased method used to examine the associations between several hundred thousand genetic polymorphisms and a particular trait. In the “Sports genetics” field, some studies have applied GWAS to identify novel variants associated with athletic performance. For sprint performance, a GWAS was conducted by Pickering et al. [41] and a related replication study by Guilherme et al. [42] identified *CPNE5* (rs3213537) as significantly associated with sprint/power athlete status. On the other hand, Ahmetov et al. [43], Rankinen et al. [44], and Al-Khelaifi et al. [45] identified several genetic polymorphisms, such as *NFIA-AS2* (rs1572312), *TSHR* (rs7144481), *RBFOX1* (rs7191721), *GALNTL6* (rs558129), and *MYBPC3* (rs1052373) as significantly associated with endurance athlete status in both GWAS discovery cohorts and replication cohorts. In addition to endurance exercise performance, Harvey et al. have performed GWAS to discover novel genetic variants associated with four weeks of high-intensity interval training (HIIT) response in the Gene SMART (Skeletal Muscle Adaptive Response to Training) study. In their study, they focused on nuclear-encoded mitochondrial proteins and identified eight polymorphisms in seven genes, *DIABLO* (rs11061368), *FAM185A* (rs113400963), *MTG2* (rs6062129 and rs6121949), *AFG3L2* (rs7231304), *NDUFAF7* (rs2041840), *TIMM23* (rs7085433), and *SPTLC2* (rs1063271), that were associated with a HIIT response [46].

### 2.3. Sports Injuries-Related SNPs

The incidence of sports injuries not only negatively influences athletic performance [47,48], but also athletic carriers as well. The idea that genetic factors partially explains the susceptibility to sports injuries was proposed a decade ago [49], and it has been suggested that genetic polymorphisms could be biomarkers for sports injuries [49,50]. Recent studies demonstrated that certain genetic polymorphisms were associated with sports injuries: *IGF2* (rs3213221), *CCL2* (rs2857656), *ELN* (rs2289360), *COL1A1* (rs1107946), *COL5A1* (rs12722), *COL22A1* (rs11784270 and rs6577958), *VDR* (rs7975232), *MCT1* (rs1049434), *ACTN3* (rs1815739), *HGF* (rs5745697 and rs1011694), *SOX15* (rs4227), *ESR1* (rs2234693), *CYP19A1* (rs936306), *ACE* (rs4341), *MMP3* (rs679620), *TNC* (rs2104772), *IL6* (rs1800795), *NOS3* (rs1799983), *HIF1A* (rs11549465) for muscle injury [51,52,53,54,55,56,57,58,59,60,61,62,63,64], *ELN* (rs2289360), *COL1A1* (rs1800012), *COL5A1* (rs12722, rs71746744, rs16399, rs4919510), *MIR608* (rs4919510) for ligament or tendon injury [51,55,65,66,67,68,69,70], and *RANK* (rs3018362), *RNAKL* (rs1021188), *OPG* (rs4355801), *P2X7R* (rs1718119 and rs3751143), *VDR* (rs10735810, rs731236), *SOST* (rs1877632), *CYP19A1* (rs936306), *COL1A1* (rs1107946) for stress fracture [60,71,72,73,74]. Therefore, these genetic polymorphisms might contribute to athletic performance by modifying susceptibilities to sports injuries. However, the research on genetics in sports injuries has just begun and the quality of the evidence might not be enough yet due to the small sample size, lack of replication, and sampling biases. For example, the sample size in most of the studies except some [59,60,61,70,72] were only a few hundred, and there was no replication cohort or additional functional analysis. Thus, future studies that address these limitations are necessary. Additionally, since these genetic polymorphisms were identified using hypothesis-based approaches, unbiased GWAS and/or whole genome sequencing for sports injuries in athletes are required to understand the details of the genetic factors for sports injuries.

## 3. Mitochondrial Genome Encoded Variants in Athletes

The mtDNA, a double-stranded circular DNA, consists of 16,569 base pairs and contains thirteen protein-coding genes, two ribosomal RNAs (rRNAs), and twenty-two transfer RNAs (tRNAs). One of the most important roles of the mitochondria is energy production. The adenosine triphosphate (ATP), necessary for skeletal muscle contraction during exercise, is produced most efficiently by the oxidative phosphorylation (OXPHOS) system in the mitochondria. Because of their roles in cellular energy production, mitochondria have been closely examined in the exercise/sports field. For example, it is well known that endurance exercise training increases mitochondrial content in the skeletal muscle and leads to enhanced exercise endurance through improving ATP production [75,76]. The 13 mtDNA-encoded proteins in the mtDNA constitute a part of the OXPHOS, and they are essential for the OXPHOS function. Indeed, it has been reported that mtDNA depletion is closely connected to the abolished OXPHOS system [77] and the sequence variations of mtDNA influence OXPHOS function in mice [78]. Therefore, it is plausible that mitochondrial single nucleotide polymorphisms (mtSNPs) regulate exercise performance in human subjects.

Several studies have demonstrated that mtSNPs influence aerobic-exercise capacity and training response to exercise training. Dionne et al. reported that three restriction fragment-length polymorphisms, such as BamHI-morph 3 in the ND5 gene (m.13470A>G), MspI-morph 4 in the gene for threonine tRNA (m.15925C>T), and NciI-morph 2 in the ND5 gene (m.13365C>T) were cross-sectionally associated with maximal oxygen uptake. Additionally, they also demonstrated that the HincII-morph 1 in the ND5 gene (m.12406G>A) was associated with a maximal oxygen-uptake response to a 20-week exercise training [79]. Nevertheless, Rivera et al. reported there were no differences in the frequencies of these mtSNPs between 125 elite endurance athletes and 65 controls (Rivera et al. 1998). A recent study by Harvey et al. applied whole-mtDNA sequencing and high-throughput genotyping arrays to identify genetic polymorphisms that are associated with exercise training responses in 62 subjects who participated in the GeneSMART study [46]. Although none of the mtSNPs passed the false discovery rate < 0.05, they found that four mtSNPs, such as m.8701A>G, m.10873T>C, m.12705C>T, and m.15043G>A, were associated with an exercise training response assessed via a change in the lactate threshold (*p*< 0.05) [46]. Additionally, Vellers et al. have suggested that several mtSNPs are associated with trainability in maximal oxygen uptake (VO2 max) to an aerobic exercise training program [80]. They sequenced the complete mtDNA sequence in the 15 highest responders and 15 lowest responders in the VO2 max response distribution of the HERITAGE (HEalth, RIsk factors, Exercise Training And Genetics) Family Study. In the lowest responders, the frequencies of the m.185G>A, m.228G>A, m.295C>T, m.462C>T, m.489T>C, m.4215T>C, m.10397A>G, m.11250A>G, m.13707G>A, m.14797T>C, m.15451C>A, m.16068C>T, and m.16125T>C were higher than the highest responders [80].

Mikami et al. analyzed the entire mtDNA sequence in 185 elite Japanese athletes and 672 controls. They found that a total of 17 mtSNPs were associated with the elite endurance/middle power or sprint/power athletes. Specifically, the m.152T>C, m.4343A>G, m.11215C>T, m.15518C>T, and m.15874A>G were associated with the elite endurance/middle-power athlete status, whereas m.151C>T, m.204T>C, m.4833A>G, m.5108T>C, m.5601C>T, m.7600G>A, m.9377A>G, m.13563A>G, m.14200T>C, m.14569G>A, m.15314G>A, and m.16278C>T were associated with the elite sprint/power athlete status.

The development of GWAS dramatically improved the detection of the genetic variants that associate with human traits. However, the mtDNA variants were exceptions to this: existing genomic pipelines are primarily designed for the nuclear DNA variants and mtDNA variants are excluded from the GWAS analysis because it does not undergo recombination or follow the Hardy–Weinberg equilibrium. Recently, PLINK, a commonly used GWAS tool, was updated for mitochondrial GWAS (MiWAS) and Miller et al. have demonstrated that including mitochondrial principal components as regression covariates could be useful for identifying mtDNA variants that associate with phenotypes in MiWAS [81,82]. Although there is no gold standard method for MiWAS yet, these improvements in the analytics pipelines will accelerate mitochondrial genetics.

## 4. Mitochondrial Microproteins: Mitochondrial-Derived Peptides (MDPs)

Although the Human Genome Project identified that there are over 20,000 genes encoding functional proteins, recent bioinformatics analyses have suggested that the human genome contains previously unannotated smORFs that might be translated into microproteins [83,84]. The term smORF was introduced to identify the ORFs with less than 100 codons that are actually translated, and the term “microprotein” refers to biologically active proteins shorter than 100 amino acids encoded in the smORFs [11,12]. Bioinformatics analysis predicts that there may be millions of theoretical microproteins in the human genome, and ribosome profiling experiments identified that there are tens of thousands of potential microprotein mRNAs [83,84,85]. However, most of them have not been detected via mass spectrometry yet because of their size, low abundance, or hydrophobicity.

**Figure 1:**
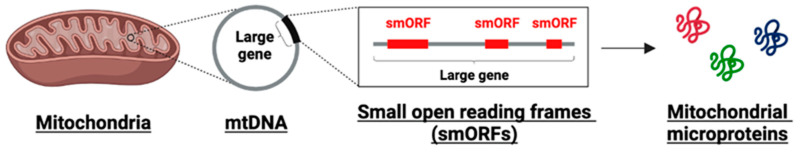
Concept of mtDNA-encoded small open reading frames (smORFs) and mitochondrial microproteins.

**Figure 2:**
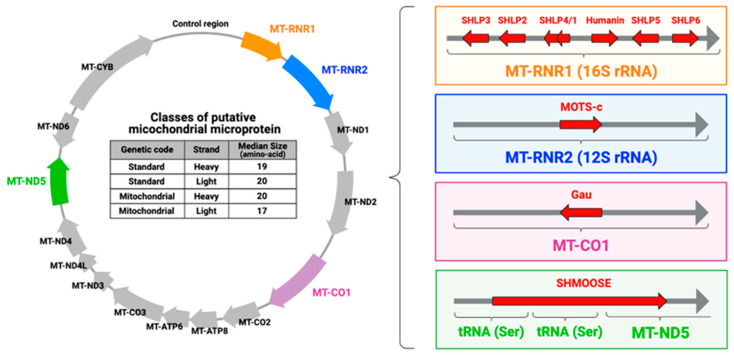
Putative and previously identified mitochondrial microproteins.

Currently, human mtDNA is annotated with 37 genes in total: thirteen protein-coding genes, two rRNAs, and twenty-two tRNAs. However, it has been demonstrated that there are dozens of previously uncharacterized cleavage sites and small RNAs derived from tRNAs with unknown functions [86], implying the existence of mitochondrial microproteins (Figure 1). Indeed, recent in silico analyses discovered that mtDNA contains nearly 400 putative microproteins between 9 and 40 amino acids length in both strands [87,88,89,90] (Figure 2). These microproteins are called mitochondrial microprotein or mitochondrial-derived peptides and nine mitochondrial microproteins, such as humanin [13,14,15], MOTS-c [16], SHLPs 1–6 [17], and SHMOOSE [18] have been identified (Figure 2). Among these mitochondrial microproteins, Humanin and MOTS-c have been studied deeply after their identification. These discoveries provided a paradigm-shifting concept in mitochondrial biology and genetics because they were previously unannotated mtDNA-encoded microproteins found to exist and have biological activities. Additionally, some of the MDPs are encoded in the mtDNA, but their translation occurs in the cytoplasm using the standard genetic code, not the mitochondrial genetic code. For example, the MOTS-c smORF is encoded within the 12S rRNA and is translated into a 16-amino acid microprotein using the standard genetic code [16]. If the MOTS-c smORF is translated using the mitochondrial genetic code, the second codon becomes the termination codon, and only the first amino acid methionine is translated. Thus, although the detailed mechanisms are not clarified yet, this suggests that a polyadenylated transcript is exported from the mitochondria and is translated in the cytoplasm. These discoveries provided novel concepts in mitochondrial biology and genetics and will give us a better understanding of human biology.

### 4.1. MOTS-c

#### 4.1.1. MOTS-c as a Metabolic Regulator

MOTS-c is a 16-amino acid microprotein encoded by a mitochondrial sORF within the 12S rRNA [16] and is expressed in several tissues including the skeletal muscle [16,91]. The first study reported by Lee et al. demonstrated that MOTS-c prevented weight gain in high-fat diet-fed mice and improved insulin sensitivity in old mice through increasing endogenous AICAR levels and activating AMPK [16]. Likewise, a separate study by Zempo and Kim et al. also reported that MOTS-c treatment and overexpression increased glucose uptake in myotubes and human embryonic kidney cells, respectively [21]. Additionally, three weeks of MOTS-c administration prevented increased body fat mass and impaired glucose uptake in high-fat diet-fed male mice, but not female mice [21]. On the other hand, it has also been suggested that MOTS-c increases the thermogenesis of white and brown fat, which also contributes to weight reduction [92,93]. Taken together, these studies suggest that MOTS-c regulates energy metabolism by improving insulin resistance in the skeletal muscle and thermogenesis in the fat [94,95].

#### 4.1.2. MOTS-c and Exercise-Related Phenotypes

Insulin resistance is not only a leading cause of obesity and type 2 diabetes but also a cause of skeletal muscle wasting and weakness. Recent studies have suggested that insulin resistance accelerates the loss of skeletal muscle mass and strength in people with type 2 diabetes [96,97,98]. Therefore, MOTS-c could prevent skeletal muscle wasting and its related signaling pathways in the skeletal muscle caused by insulin resistance. Three weeks of MOTS-c administration significantly prevented skeletal muscle loss and myostatin mRNA expression, one of the strongest negative regulators of the skeletal muscle, in high-fat diet-fed mice [99]. Additionally, the study observed that MOTS-c regulated the CK2/PTEN/AKT/FOXO1 signaling pathway in the skeletal muscle, and a negative correlation between plasma MOTS-c and myostatin levels in human plasma [99]. Supporting this observation, Reynolds et al. demonstrated that long-term MOTS-c-treated middle-aged and old mice exhibited higher lean mass and muscular strength than the control groups [100]. Taken together, MOTS-c could be a potential target for regulating skeletal muscle mass through modifying classical muscle atrophy signaling and myostatin expression.

**Figure 3:**
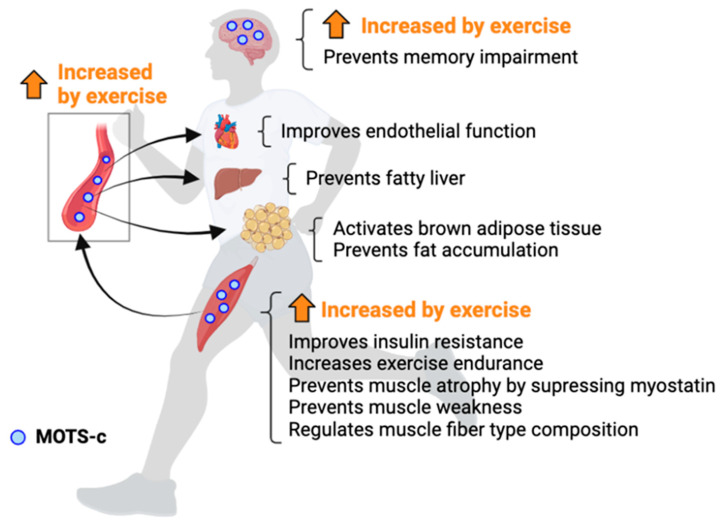
The effects of MOTS-c in systemic organs.

In terms of the association between MOTS-c and exercise, several studies have demonstrated that MOTS-c is an exercise-induced and exercise-mimetic microprotein. Reynolds et al. examined the effect of acute cycling exercise on the MOTS-c level in the skeletal muscle and plasma and observed that acute high-intensity interval exercise increased MOTS-c expression in both the skeletal muscle and plasma in young male subjects [100]. Although it was not statistically significant, Walden et al. also observed that acute aerobic exercise increased plasma MOTS-c levels by around 30–40% compared to prior to exercise [101]. Dieli-Conwright et al. demonstrated that a 16-week combination training of aerobic and resistance exercise increased plasma MOTS-c levels in Non-Hispanic breast cancer survivors [102]. An increased MOTS-c expression by exercise training was also observed in animal experiments [103,104]. Hyatt J.K. and Kang et al. reported that running exercise training increased MOTS-c expression in the rat skeletal muscle and mouse hypothalamus, respectively [103,104]. On the other hand, however, Ramanjaneya et al. showed that MOTS-c did not increase after 8-week aerobic exercise training in women with polycystic ovarian syndrome [105]. Differences in a study subject, exercise protocol, detection method, and/or sampling timing may cause these different observations. Although more research is necessary to confirm these observations, exercise likely increases MOTS-c expression in not only skeletal muscle but also in other tissues (Table 1 and Figure 3).

It has been suggested that MOTS-c has an exercise-mimetic effect and improved aerobic exercise performance. Reynolds et al. have demonstrated that a long-term MOTS-c administration into young and old mice significantly increased the running time and distance compared to the control mice [100]. Hyatt J.K. has confirmed this observation by a single MOTS-c administration experiment with a cross-over design [103]. Consistent with the findings reported by Reynolds et al., the running time and distance were significantly higher in the MOTS-c-administrated trial than the saline-administrated trial, and all of the examined mice exhibited an improvement in their exercise performance compared to the saline-administrated trial [103]. These studies suggest that MOTS-c clearly increases aerobic exercise performance, and a long-term administration has an aerobic exercise training-like effect in mice. Additionally, since MOTS-c is induced by exercise, MOTS-c may also be associated with a response to exercise stress. Indeed, although MOTS-c mainly localizes in the mitochondria, MOTS-c translocates from mitochondria to the nucleus following metabolic stress and regulates the nuclear DNA-encoded genes involved in oxidative stress response by interacting with the nuclear-factor erythroid 2-related factor 2 (NRF2) [106]. Therefore, MOTS-c is an exercise-induced and exercise-mimetic microprotein and contributes to aerobic exercise performance (Table 1 and Figure 3).

A recent study showed that a MOTS-c analogue has the potential to prevent cognitive decline induced by the amyloid beta (Aβ) or LPL [107]. Although the peripheral administration of MOTS-c did not cross the blood–brain barrier, administration of the cell-penetrating MOTS-c analogue significantly prevented memory impairment by suppressing neuroinflammation [107]. Since Kang et al. demonstrated that exercise training increased MOTS-c expression in the hypothalamus, upregulation of MOTS-c in the brain could be one of the underlying mechanisms of improvement in cognitive function by exercise.

#### 4.1.3. Genetic Polymorphism in the MOTS-c Coding Region

**Figure 4:**
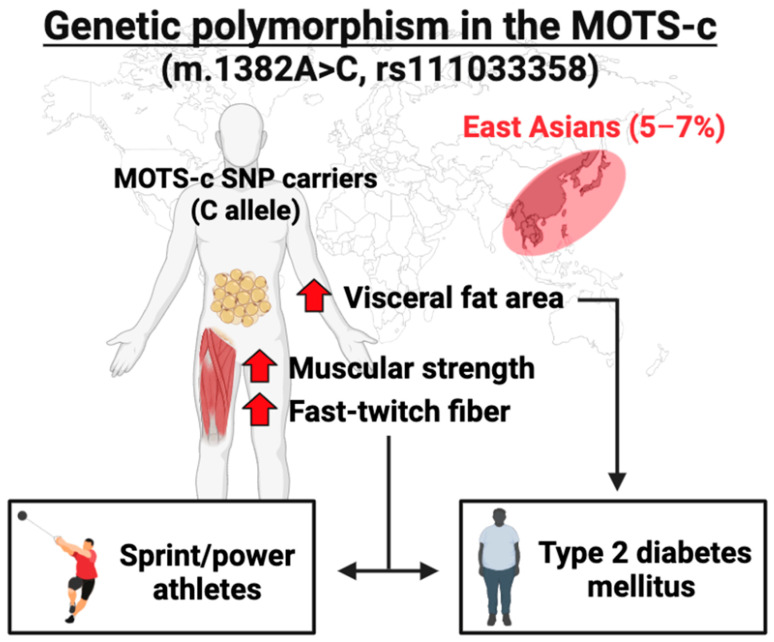
Genetic polymorphism in the MOTS-c coding region and its related phenotypes.

In the MOTS-c coding region, there is an East Asian-specific genetic variant m.1382A>C (rs111033358), and this mutation causes amino acid replacement, from positively charged lysine (K) to neutral glutamine (Q) at the 14th residue of the MOTS-c (K14Q). Zempo and Kim et al. conducted a series of experiments to understand the biological function of this K14Q mutation [21]. A meta-analysis in 11,224 Japanese males demonstrated that the males with the K14Q mutation exhibited a 1.34 times higher risk of type 2 diabetes mellitus (T2DM) as well as higher visceral fat area than the wild type (WT) carriers [21] (Figure 4). Additionally, the WT MOTS-c administration prevented impaired glucose metabolism in high-fat diet-fed mice, while K14Q failed to improve impaired glucose metabolism induced by a high-fat diet, suggesting that K14Q-MOTS-c is a bio-inactive form of MOTS-c [21] (Table 1). Interestingly, this amino acid replacement is predicted to change the charge and hydrophobicity of the MOTS-c, which could substantially alter the interactions with its binding partners [21]. Although the functional direct molecular target of MOTS-c has not been identified yet, it is expected that the WT MOTS-c and K14Q MOTS-c differentially interact with the binding partners and show different biological functions.

This K14Q mutation is one of the genetic markers for athletic performance in the East-Asian population. Among the Japanese population, the frequency of the K14Q mutation is 2.9% in endurance athletes, 5.1% in non-athlete controls, and 6.5% in sprint/power athletes, suggesting that the K14Q is more beneficial for sprint/power performance than the WT carriers [22] (Figure 4). To confirm this observation, additional analyses in two independent Japanese cohorts were conducted and they demonstrated that the K14Q mutation carriers exhibited higher muscular strength as well as a higher proportion of myosin heavy chain (MHC)-IIX than the WT carriers [22]. Furthermore, mice treated with the MOTS-c neutralizing antibody, mimicking the K14Q carriers, exhibited a significantly higher protein expression of MHC-fast than the control mice [22] (Table 1). The possible underlying mechanisms are the proliferation-activated receptor co-activator 1 (PGC-1α) and FOXO1 expression levels because they partially regulate muscle fiber-type composition. It has been demonstrated that overexpression and knock-out of PGC-1α in mice increase slow- and fast-twitch fibers, respectively [108,109]. Additionally, muscle-specific overexpression of FOXO1 decreased slow-twitch fiber-related gene expression levels [110]. Interestingly, recent studies demonstrated that MOTS-c treatment increased PGC-1α protein expression in the C2C12 myotube [111] and decreased FOXO1 protein expression in the mouse skeletal muscle [99]. These studies suggest that MOTS-c could regulate muscle-fiber composition by modifying PGC-1α and FOXO1 expression levels. Taken together, the m.1382A>C polymorphism causing K14Q amino acid replacement of MOTS-c contributes to sprint/power performance by regulating skeletal muscle fiber composition in the East-Asian population (Figure 4).

**Table 1:** Summary of published mitochondrial microprotein studies-related to exercise and muscle function.

	Stimulation	Term	Observation	Tissue	Reference
MOTS-c	MOTS-c	Chronic	Improves insulin resistance	Mouse muscle	Lee et al. [16]
	MOTS-c	Chronic	Improves glucose metabolism	Mouse muscle	Zempo and Kim et al. [21]
	K14Q MOTS-c	Chronic	No difference	Mouse muscle	Zempo and Kim et al. [21]
	MOTS-c	Acute	Increases exercise endurance	Mouse muscle	Hyatt. [103]
	MOTS-c	Chronic	Increases exercise endurance	Mouse muscle	Reynolds et al. [100]
	MOTS-c	Chronic	Prevents muscle wasting	Mouse muscle	Reynolds et al. [100]
	MOTS-c	Chronic	Improves muscle strength	Mouse muscle	Reynolds et al. [100]
	MOTS-c	Chronic	Supresses myostatin expression	Mouse muscle	Kumagai et al. [99]
	MOTS-c	Chronic	Prevents muscle wasting	Mouse muscle	Kumagai et al. [99]
	MOTS-c antibody	Chronic	Increases myosin heavy chain-fast	Mouse muscle	Kumagai et al. [22]
	AEx	Chronic	Increases MOTS-c	Mouse hypothalamus	Kang et al. [104]
	AEx	Chronic	Increases MOTS-c	Rat muscle	Hyatt. [103]
	HIIT	Acute	Increases MOTS-c	Human muscle and blood	Reynolds et al. [100]
	AEx	Acute	No difference	Human blood and muscle	von Walden et al. [101]
	REx	Acute	No difference	Human blood and muscle	von Walden et al. [101]
	AEx and REx	Chronic	Increases MOTS-c	Human blood	Dieli-Conwright et al. [102]
	AEx	Chronic	No difference	Human blood	Ramanjaneya et al. [105]
Humanin	Humanin	Chronic	Improves rotarod performance	Mouse muscle	Kim et al. [112]
	Contraction	Acute	Increases humanin	Isolated mouse muscle	Woodhead et al. [113]
	HIIT	Acute	Increases humanin	Human muscle and blood	Woodhead et al. [113]
	AEx	Acute	Increases humanin	Human blood	von Walden et al. [101]
	REx	Acute	No difference	Human blood and muscle	von Walden et al. [101]
	REx	Chronic	Increases humanin in muscle	Human muscle and blood	Gidlund et al. [114]
	HIIT	Chronic	No difference	Human muscle and blood	Woodhead et al. [113]
	AEx	Chronic	No difference	Human muscle and blood	Gidlund et al. [114]
SHLP2	HIIT	Acute	No difference	Human blood	Woodhead et al. [113]
	HIIT	Chronic	No difference	Human blood	Woodhead et al. [113]
SHLP6	HIIT	Acute	Increases SHLP6	Human blood	Woodhead et al. [113]
	HIIT	Chronic	No difference	Human blood	Woodhead et al. [113]

MOTS-c Ab: MOTS-c neutralizing antibody, AEx: aerobic exercise, REx: resistance exercise, HIIT: high-intensity interval training.

### 4.2. Humanin

#### 4.2.1. Biological Functions of Humanin

The first MDP to be discovered was humanin. Humanin is a 24-amino acid microprotein encoded within the 16S rRNA region of mtDNA and it was discovered by three independent groups in the early 2000s [13,14,15]. Hashimoto et al. initially identified humanin during a screening for genes protective against Aβ toxicity [13,115]. Also, Ikonen et al. and Guo et al. found that humanin bound IGFBP3 and BAX, and reduced Aβ toxicity and cell apoptosis, respectively [14,15]. After the identification, a trimeric receptor consisting of WSX-1, GP130, and CNTFR as well as a separate FRPL1 were identified as Humanin’s receptors [116,117], and it was demonstrated that humanin modified mitochondrial biology, cell proliferation, and cell survival by activating downstream STAT3 and ERK1/2 [118]. Hundreds of additional studies are published so far and humanin has been described as a cytoprotective and neuroprotective factor [87,88,89,90]. Since humanin modifies mitochondrial function, humanin also has beneficial effects on energy metabolism, such as preventing high-fat diet-induced weight gain, fat accumulation, increasing insulin sensitivity, and glucose stimulated-insulin release [119,120,121].

#### 4.2.2. Humanin and Exercise-Related Phenotypes

Regarding the association between humanin and skeletal muscle function, Kim et al. have demonstrated that humanin administration into aged mice improved the average running time during the rotarod performance test by increasing autophagy in the skeletal muscle [112]. Although the experimental model was the aged mouse, this study proposed that increasing humanin levels had a beneficial effect on skeletal muscle function in vivo. On the other hand, several studies have examined the effects of exercise stimulation on humanin expression in skeletal muscle and blood samples. Woodhead et al. have demonstrated that muscle contraction in isolated mouse skeletal muscle dramatically increased humanin levels four-fold, suggesting that exercise-induced muscle contraction, not exercise-induced metabolites, induces humanin expression [113]. Inconsistent with this observation, acute high-intensity interval exercise and aerobic exercise upregulated humanin expression in human skeletal muscle and/or plasma, while acute resistance exercise did not change plasma humanin levels [101,113]. In terms of chronic exercise training, although Gidlund et al. observed that resistance exercise training increased humanin expression only in the skeletal muscle, other studies did not observe increased humanin expression [113,114]. A possible explanation for these inconsistent findings is baseline humanin levels. Humanin levels are downregulated in people with metabolic disorders [114,122], and the study subjects in Gidlund et al. were males with impaired glucose metabolism [114], suggesting that baseline humanin levels were lower compared to other subjects. Another explanation is sampling timing. Because of the amino acid length, the half-life of humanin is shorter than 30 min [123]. Thus, sampling timing is also an important factor that needs to be considered. These could be possible explanations for the inconsistent results in the exercise response. Altogether, acute aerobic exercise upregulates humanin expression, while chronic exercise training may not change humanin expression. Further studies are required to identify humanin regulation by exercise.

#### 4.2.3. Genetic Polymorphism in the Humanin Coding Region

A naturally occurring m.2706A>G polymorphism (rs2854128) on the humanin coding region is associated with decreased circulating humanin levels [20]. Furthermore, this mutation accelerates cognitive aging in African Americans [20]. Although this genetic variant does not change the amino acid sequence of humanin, these data suggest that this mutation is functional and influences phenotypes in human subjects. Interestingly and on the other hand, one of the humanin receptors is CNTFR, and Miyamoto-Mikami et al. have demonstrated that the genetic variant in the CNTFR (rs41274853) was associated with sprint/power exercise performance [124]. Thus, there may be an interaction between genetic polymorphisms in humanin and CNTFR. Therefore, it might be interesting to examine the association of this humanin variant and exercise performance as well as the interaction between humanin and CNTFR.

### 4.3. SHLPs

After the discovery of humanin, six additional mitochondrial microproteins encoded around humanin smORF were identified and named small humanin-like peptides 1 to 6 (SHLPs 1–6) because they were encoded from the 16S rRNA region and share some biological features with humanin, modulating mitochondrial function and decreasing Aβ toxicity [17]. SHLP2, one of the SHLPs, shows protection against Aβ-induced toxicity similar to that of humanin [125]. It has also been suggested that both SHLP2 and humanin analog have chaperone-like activity by targeting the misfolding of islet amyloid polypeptide (IAPP), a critical pathogenic step in T2DM, and inhibit IAPP misfolding [126]. The effect of SHLP2 on metabolic function has been examined in vitro and in vivo [17]. SHLP2 treatment promotes mitochondrial biogenesis, reduces reactive oxygen species, and decreases mtDNA oxidation. In addition, in the presence of insulin, SHLP2 accelerates the differentiation of 3T3-L1 pre-adipocyte by supposedly increasing insulin sensitivity [17]. Furthermore, intracerebroventricular (ICV) infusion of SHLP2 increases insulin responsiveness as assessed by the exogenous glucose infusion rate and suppression of hepatic glucose production and peripheral glucose uptake under systemic pancreatic insulin clamp and physiologic hyperinsulinemic-euglycemic clamp studies [17]. Therefore, these studies suggest that SHLP2 has potential as a metabolic therapeutic as well as a regulator of exercise training response.

Regarding the effect of exercise on SHLP levels, one study examined the effect of acute high-intensity interval exercise and HIIT on SHLP2 and SHLP6 levels [113]. They observed that acute high-intensity interval exercise increased plasma SHLP6 levels, while HIIT decreased plasma SHLP6 levels. SHLP2 did not show any differences before and after the exercise [113]. Further studies are necessary to discuss about exercise and SHLPs.

### 4.4. SHMOOSE

A mitochondrial microprotein called SHMOOSE was identified using several detection methods [18]. Two unique SHMOOSE fragments were identified in mitochondria fractions using mass spectrometry. In addition, SHMOSE was detected in human cerebrospinal fluid using ELISA, and its levels correlated to age and the Alzheimer’s disease biomarker phosphorylated tau. SHMOOSE was targeted because its smORF contains a common single nucleotide polymorphism towards the 3’ region (m.12372G>A, rs2853499), causing a missense mutation at the 47th amino acid (SHMOOSE.D47N). Individuals with rs2853499 had a 30% greater risk for AD and accelerated brain atrophy and hypometabolism in regions in brain vulnerable regions. SHMOOSE.D47N affects the predicted disordered C-terminus of SHMOOSE, leaving its predicted amphipathic alpha helical feature unaffected. Many other microproteins also have highly disordered regions that promote a higher assembly of protein complexes, such as proteins within mitochondria. Indeed, SHMOOSE binds the inner mitochondrial membrane mitofilin, increases mitochondrial spare capacity, and reduces mitochondrial superoxide production. When mitofilin is knocked down with siRNA, the effect of SHMOOSE on mitochondrial superoxide is muted. Furthermore, neuronal cells stressed with amyloid beta oligomers and simultaneously exposed to SHMOOSE confer protection, while SHMOOSE.D47N did not confer protection. Altogether, SHMOOSE could be involved in AD pathology via its biological effects within the inner mitochondrial membrane.

### 4.5. Gau

Gau is an approximately 100-amino acid mitochondrial microprotein encoded within the MT-CO1 region [19]. The Gau protein sequence is relatively well conserved in protist, fungal, plant, and animal mtDNAs, suggesting that Gau has a possible conserved biological function. However, to date, its biological function has not been clarified yet. Immunohistochemical analysis using an anti-Gau antibody demonstrated that Gau mainly localized in the mitochondria. Thus, although those observations suggest that Gau has crucial biological roles in the mitochondria, further studies are necessary to address a biological function of Gau.

## 5. Implication of Sports Genetics in Age-Related Diseases

Although the genetics in athletes and aging-related diseases and disorders do not seem to be related at a glance, studies have suggested a connection between them. For example, skeletal muscle fiber composition, largely influenced by genetic factors, affects glucose uptake [127,128] because each fiber type has specific characteristics; slow-twitch fibers contain high levels of oxidative enzymes and low levels of glycolytic enzymes, whereas fast-twitch fibers contain high levels of glycolytic enzymes and low levels of oxidative enzymes [129]. The fast-twitch fibers are suitable for sprint/power performance [130], while the proportion of fast-twitch fiber is negatively correlated to glucose uptake [127,128]. Indeed, a high proportion of fast-twitch fibers causes metabolic disorders, such as obesity and metabolic syndrome [131,132]. One example is the MOTS-c polymorphism (m.1382A>C, rs111033358). The K14Q mutation carriers of the MOTS-c polymorphism exhibit a higher proportion of fast-twitch fibers than those with WT, and the frequency of this mutation is higher in the sprint/power athletes than others [22], while the risk of T2DM is significantly higher in the K14Q mutation carriers than the WT carriers [21]. Thus, although people with the MOTS-c variant have an advantage in sprint/power performance, the risks of metabolic disorders are also high in mutation carriers. Similar to metabolic disorders, muscle fiber composition also influences blood pressure [133,134]. A 19-year follow-up study demonstrated that the proportion of slow-twitch fibers is negatively associated with systolic and diastolic blood pressure [134], implying that muscle fiber composition-related genetic variants are candidates for genetic biomarkers for hypertension. Taken together, future studies that connect the findings between sports science and medicine are interesting and necessary.

## 6. Perspective: Future Directions of mtDNA and Microproteins

mtDNA sequences are more varied among ethnicities compared to the nuclear DNA due to its high mutation rate [81]. Thus, mtDNA variants could be an ethnicity-specific genetic marker for athletic performance as well as age-related diseases. Given the improvements in the analytics pipelines for mtDNA, future studies will identify ethnicity-specific mtDNA variants that could explain health disparities in ethnicities as well as exercise performance. In addition, although mtDNA has been considered to encode 13 protein-coding genes, it may encode hundreds of uncharacterized mitochondrial microproteins, and these microproteins could be easily accessible biomarkers and therapeutic targets [87,88,89,90]. Over twenty years have passed since the first mitochondrial microprotein humanin was discovered, but this field is still developing and additional microproteins have been identified these days. Although there will still be technical challenges, such as identification and detection, this novel field is one of the most promising topics [135] and should be investigated to unravel hidden human biology.

## 7. Conclusions

In summary, mitochondrial microproteins are encoded in the smORF within the mtDNA, and ten mitochondrial microproteins, such as humanin, MOTS-c, SHLPs 1–6, SHMOOSE, and Gau have been identified to date. Among them, MOTS-c is a leading mitochondrial microprotein in terms of a regulator of skeletal muscle function, and genetic variant in the MOTS-c coding region is associated with athletic performance as well as type 2 diabetes by modulating skeletal muscle properties. Future studies are expected to identify the roles of mitochondrial microproteins in human biology, including skeletal muscle biology.
